# PFKP Mediates Breast Cancer Metastasis Through Altered Glycolysis

**DOI:** 10.7759/cureus.87880

**Published:** 2025-07-14

**Authors:** Summayya Anwar, Farhan Haq, Muhammad Saeed

**Affiliations:** 1 Biosciences, Commission on Science and Technology for Sustainable Development in the South (COMSATS) University Islamabad, Islamabad, PAK

**Keywords:** breast cancer, hypoxia, metabolism, metastasis, phosphofructokinase (pfkp)

## Abstract

Background

Despite recent breakthroughs in genetic profiling, breast cancer metastasis remains a considerable challenge affecting treatment and overall patient survival. Therefore, the discovery of target alternatives to restrain metastasis is urgently needed. In the current study, we aimed to identify novel targets driving metastasis and elucidate the underlying mechanisms.

Methods

We initially identified differentially expressed genes between primary breast tumors and metastatic breast cancer patients using datasets from the Gene Expression Omnibus (GEO) database. Subsequently, we validated these findings by examining the changes in gene expression and their direction in external datasets. Furthermore, we identified the significantly enriched pathways associated with gene expression. We analyzed PFKP expression patterns in 100 samples (normal, primary breast tumor, and metastasis) using quantitative real-time polymerase chain reaction (qRT-PCR), and survival analyses were performed.

Results

We identified 34 differentially expressed genes in metastatic breast tumors, with CCDC6, PKIA, UACA, and PFKP significantly upregulated (p < 0.05). PFKP was highly expressed in metastasis, negatively correlated with ER/PR/HER2 status, and linked to glycolysis-related genes (ENO1, PGM1, LDHB, and PGK1). Gene set enrichment analysis (GSEA) highlighted its role in glucose metabolism, hypoxia, and angiogenesis. qRT-PCR confirmed PFKP (p < 0.001) and Ki67 (p < 0.001) upregulation in 100 breast cancer samples. PFKP correlated with Ki67, and receiver operating characteristic (ROC) analysis (area under the curve (AUC) > 71%) indicated a strong predictive value. Higher PFKP expression was associated with poor survival, supporting its role as a prognostic marker.

Conclusion

The current study showed that PFKP promotes tumor metastasis through hypoxia-mediated altered glycolysis and can be a potential prognostic marker used to identify breast cancer metastasis.

## Introduction

Breast cancer (BC) has the highest incidence and mortality rate worldwide [1]. According to the WHO, 2.3 million BC cases were diagnosed in 2020, and 685,000 deaths were reported in women [2]. Metastasis is the most notable clinical outcome of invasive BC. Studies show that 20%-30% of patients with primary breast tumors develop metastasis, contributing to 90% of BC-related mortality [3]. Therefore, it is crucial to identify the genetic factors involved in metastatic transformation to improve overall survival (OS) rates in cancer patients and to design novel therapies that can reduce BC metastasis and, consequently, the overall disease burden.

Metabolic reprogramming is an imperative feature of malignant tumors, enabling them to invade (metastasize) neighboring tissues and organs. Since cancer cells show increased glucose uptake, cells' growing energy demands lead to considerable alterations in glycolysis under normoxic conditions. Thus, the characteristic shift in cellular respiration under these conditions reflects the Warburg effect, known as aerobic glycolysis [4]. Notably, the genes involved in metastasis may not confer enhanced proliferative benefit to the primary tumor [5]. This striking behavior of metastatic genes has made their identification difficult. However, several genetic studies have shown potential metastatic genes in patients with primary BC (PBC) [6]. A comprehensive in silico analysis of available data might show and evaluate potential metastatic markers with prognostic significance. A few of the known metastatic gene signatures include the MammaPrint (a 70-gene signature), Oncotype (a 21-gene signature), and PAM50 [7]. The PAM50 prediction analysis demonstrates potential metastatic cases in ER+ BC patients. However, it has limited implications as it can reveal patients with a reduced risk of distant recurrence and Ki67 expression signatures in a similar study cohort [8].

A rate-limiting enzyme of glycolysis is called PFKP, which is essential for the metabolic reprogramming observed in cancer cells. This enzyme facilitates the phosphorylation of fructose-6-phosphate into fructose-1,6-bisphosphate, a key regulatory step within the glycolytic pathway. The overexpression of PFKP in cancer cells promotes increased glucose uptake and enhanced glycolysis, providing the necessary energy and metabolic substrates for tumor growth and survival. Previously reported studies showed the upregulation of PFKP in different cancers. However, its role in metastasis in BC is still unclear.

This study aims to analyze the alterations in gene expression profiles associated with the metastatic transformation of primary breast tumors. For this purpose, integrative analysis discovered differentially expressed gene (DEG) signatures in 463 primary and metastatic tumors in BC patients. Furthermore, in silico approaches were used to evaluate the functional significance of biochemical pathways, elucidating the roles of these genes in metastatic transformation. The identified DEGs were significantly associated with these pathways, revealing a potential gene network involved in activating the metastatic cascade. This study highlights PFKP as a novel candidate gene in the metastatic progression of breast tumors, which may have prognostic and therapeutic relevance in breast carcinogenesis.

## Materials and methods

Data collection and processing

We retrieved discovery cohorts 1 and 2 (GSE43837 and GSE58984) from the Gene Expression Omnibus (GEO) database. The datasets comprise the Affymetrix (GSE43837-Affymetrix Human X3P Array and GSE58984-Affymetrix Human Genome U133 Plus 2.0 Array) platforms. Discovery cohort 1 (GSE43837 dataset) includes expression data from 38 patients, 19 of which were metastatic and 19 were primary breast tumor samples. Meanwhile, discovery cohort 2 (GSE58984) contains expression data from 94 samples, including 11 metastatic models and 83 primary mammary tumors. The clinicopathological parameters of discovery cohorts 1 and 2 are summarized in Table 1.

**Table 1:** Clinicopathological parameters of discovery and validation cohorts 1 & 2. N: sample number; M: mean; M0: no metastasis; M1: metastasis.

Parameters	Discovery cohort 1 sample n = 38	Discovery cohort 2 sample n = 94	Validation cohort 1 sample n = 66	Validation cohort 2 sample n = 265
Metastasis stage				
M0 (primary tumor)	19	83	18	210
M1 (metastasis)	19	11	13	55
Age category				
≤50 years	14	42	13	103
≥50 years	24	52	18	161
ER status (estrogen receptor)				
ER-positive	8	60	9	49
ER-negative	30	34	22	216
PR status (progesterone receptor)				
PR positive	-	47	27	42
PR negative	-	47	16	223
Tumor size				
T1 & T2 (<5 cm)	-	0	-	35
T3 & T4 (>10 cm)	-	94	-	159
Tumor grade				
I	-	-	10	80
II & III	-	-	19	126
Nodal involvement				
N0	-	91	-	-
N1	-	3	-	-
Ki67%				
<20	-	-	27	-
>20	-	-	16	-

**Figure 1:**
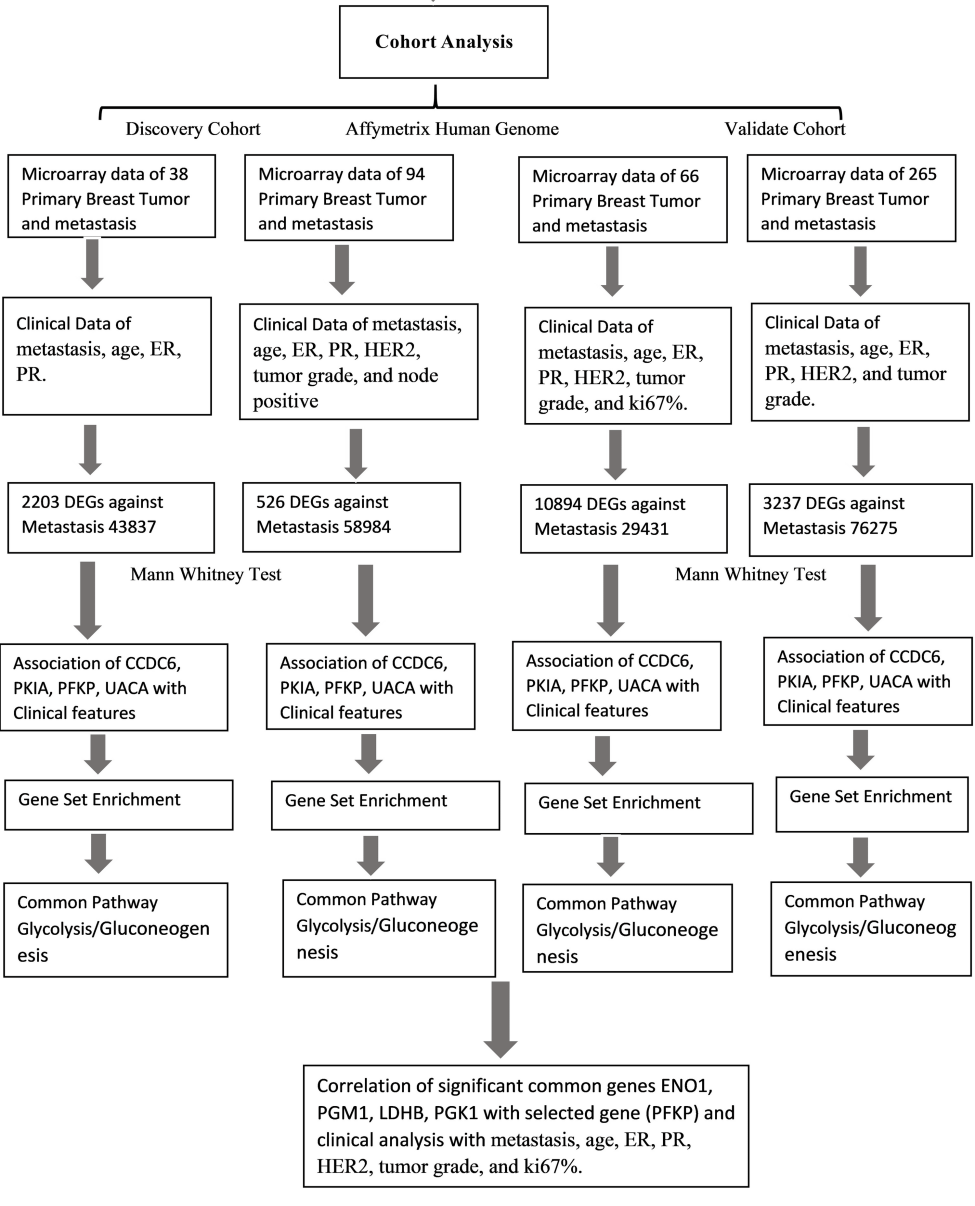
Workflow of the cohort analysis performed in discovery and validation datasets. This schematic represents the study design and analytical workflow involving four breast cancer cohorts. The discovery cohorts (GSE43837 and GSE58984) and validation cohorts (GSE29431 and GSE76275) include microarray-based gene expression data of primary breast tumors and metastatic samples. Each dataset was subjected to differentially expressed gene (DEG) analysis against metastasis using the Mann-Whitney U test. Genes CCDC6, PKIA, PFKP, and UACA were identified as common significant genes across all cohorts and were further analyzed for correlation with clinical parameters. Gene set enrichment analysis (GSEA) revealed glycolysis/gluconeogenesis as a commonly enriched pathway. Correlation analysis was performed for key glycolysis genes (ENO1, PGM1, LDHB, and PGK1) and PFKP in association with clinical features. ER: estrogen receptor; PR: progesterone receptor; HER2: human epidermal growth factor receptor 2; CCDC6: coiled-coil domain containing 6; PKIA: protein kinase inhibitor alpha; PFKP: phosphofructokinase, platelet isoform; UACA: uveal autoantigen with coiled-coil domains and ankyrin repeats; ENO1: enolase 1; PGM1: phosphoglucomutase 1; LDHB: lactate dehydrogenase B; PGK1: phosphoglycerate kinase 1; Ki67%: percentage of Ki67-positive cells indicating proliferative activity.

We also retrieved microarray data GSE29431 of 66 primary and metastatic BC patients from the GEO database to validate the findings of the discovery cohort. Cohort 3 (GSE29431-Affymetrix Human Genome U133 Plus 2.0 Array) included 66 primary and metastasis breast tumor samples and details of their clinicopathological attributes such as metastasis, age, ER and PR, HER2, tumor grade, and Ki67% (Table 1). Cohort 4 (GSE76275-Affymetrix Human Genome U133 Plus 2.0 Array) included 265 primary and metastatic tumor samples and their clinicopathological attributes (Table 1). The overall study design is presented in Figure 1. The current study found the DEGs of each dataset using BRB-Array Tools version 4.6.1. Gene fold change (FC) represents the relationship between gene expression levels for metastasis and the primary breast tumor.

DEG analysis

To assess differential gene expression between primary breast tumors and metastatic samples, we utilized BRB-Array Tools version 4.6.1 [9], a comprehensive platform designed for microarray data visualization and statistical evaluation. Genes exhibiting an FC above 1.5 and a p-value below 0.05 were classified as DEGs in this study.

Subsequently, the overlapped DEGs, as well as upregulated and downregulated genes, from these four datasets were analyzed using Venny 2.1 software [10]. Upregulated genes were selected for further analysis due to their targetability.

Gene network functional analysis

GSEA was conducted using the GSEA software from the Broad Institute [11] to demonstrate potential gene networks related to the PFKP gene. For this analysis, the samples from the datasets GSE29431 and GSE58984 were classified into two groups based on their PFKP expression levels: high and low. We used the Molecular Signatures Database (MSigDB) to assess enrichment in Gene Ontology (GO) [12]. The criteria for significant enrichment were a normalized enrichment score (NES) |NES| > 1 and a false discovery rate (FDR)-q-value < 0.05, consistent with standard GSEA thresholds for significance. The pathway was generated using Cytoscape.

Patient and sample collection

We analyzed PFKP expression in approximately 100 tissue samples and Ki67 expression in 30 fresh tissue biopsy samples. These specimens were collected from patients with histopathologically confirmed metastatic and primary breast tumors, as well as adjacent normal control tissues (ANCTs), during surgery at local hospitals in Rawalpindi and Islamabad (Holy Family and Pakistan Institute of Medical Sciences) after obtaining signed informed consent (ethical approval reference: CUI/Bio/ERB/2017/20). The collected biopsy samples were stored immediately after surgery in RNA Applicable later solution at -80°C until further use. The clinicopathological characteristics and association of PFKP are mentioned in Table 2.

**Table 2:** Association of clinicopathological characteristics with PFKP expression in control and tumor samples. * shows the significance of tumor vs. control with p-value < 0.05, ** shows significance at p < 0.01, and **** shows significance at p < 0.0001. Statistical comparisons were performed using the Mann-Whitney U test (for overall tumor vs. control), independent t-tests (for binary group comparisons), and one-way ANOVA (for comparisons involving more than two groups). PFKP: phosphofructokinase, platelet isoform; N: sample number; M: mean expression; t: Student’s t-test value; F: F-statistic from ANOVA; DCIS: ductal carcinoma in situ; LCIS: lobular carcinoma in situ; IDC: invasive ductal carcinoma; ILC: invasive lobular carcinoma; M0: no metastasis; M1: metastasis present.

Variables	PFKP expression		p-value	U/t/F-value
	Control N (M)	Tumor N (M)	<0.0001****	U = 3,308
Overall status	100	100		
Age (years)				
<50	52 (0.98)	52 (1.52)	0.553	t = 11.28
≥50	48 (1.02)	48 (1.44)		
Tumor size (cm)				
<2	11 (0.69)	11 (1.73)	0.582	F = 0.54
2-5	50 (0.99)	50 (1.56)		
>5	39 (1.09)	39 (1.32)		
Clinical stages				
I	17 (0.66)	17 (1.50)	0.0421*	F = 2.84
II	30 (1.04)	30 (1.65)		
III	29 (1.18)	29 (1.07)		
IV	24 (0.96)	24 (1.77)		
Tumor grade				
I	12 (0.67)	12 (1.04)	0.2245	F = 1.52
II	55 (0.98)	55 (1.58)		
III	33 (1.13)	33 (1.48)		
Carcinoma histology				
DCIS	25 (0.98)	25 (1.13)	0.0644	F = 2.50
LCIS	06 (0.69)	06 (2.08)		
IDC	39 (1.04)	39 (1.44)		
ILC	30 (1.02)	30 (1.72)		
Nodal involvement				
Positive	61 (1.07)	61 (1.57)	0.3509	t = -0.94
Negative	39 (0.88)	39 (1.36)		
Metastasis				
M0-primary tumor	71 (0.89)	71 (1.33)	0.01688**	t = -2.46
M1-metastasis	29 (1.25)	29 (1.86)		
Menopausal				
Pre	57 (0.95)	57 (1.42)	0.5356	t = -0.62
Post	43 (1.06)	43 (1.57)		
Treatment (chemotherapy)				
Yes	16 (0.98)	16 (1.70)	0.3617	t = 0.93
No	84 (1.003)	84 (1.44)		

RNA extraction and real-time polymerase chain reaction quantification

Total RNA was extracted from tissue samples using the standard Trizol protocol with minor modifications, as previously described [13]. RNA concentration and purity were assessed using a Nanodrop spectrophotometer by measuring absorbance ratios at 260/280 and 260/230 nm, supplemented by visualization on 1% agarose gel electrophoresis. Complementary DNA (cDNA) synthesis was performed via reverse transcription polymerase chain reaction (RT-PCR) using the Superscript First-Strand cDNA Synthesis Kit (Invitrogen, Carlsbad, CA, USA). Quantitative real-time PCR (qRT-PCR) was conducted using gene-specific primers for PFKP, Ki67, and β-actin as the internal control (Supplemental material 1). Amplification was carried out on a Step One Plus Real-Time PCR System (Applied Biosystems, Waltham, MA, USA). Thermal cycling conditions included an initial denaturation at 95°C for two minutes, followed by 40 cycles of 95°C for 15 seconds, 54°C for 60 seconds, and 72°C for 20 seconds, with a final extension at 72°C for one minute. Expression levels were analyzed using the 2^-ΔΔCT^ method [14].

Survival analysis

The study employed the Kaplan-Meier method, a non-parametric method that evaluates the survival function using censored data. This approach was used to generate survival curves for both PFKP Exp downregulation and upregulation, and metastatic and primary breast tumor patients. The median survival time was determined for each group, and 95% confidence intervals were computed using R version 4.1.1 (R Foundation for Statistical Computing, Vienna, Austria). A log-rank (Mantel-Cox) test was performed to assess statistical significance and compare survival curves between the groups.

Correlation analysis and receiver operating characteristic analysis

Correlation analysis was conducted to investigate the relationship between candidate genes. The results revealed significant associations between candidate genes. Additionally, GraphPad Prism 9.0 software (Dotmatics, Boston, MA, US) was used for a receiver operating characteristic (ROC) analysis to assess the diagnostic accuracy of the proposed model, yielding promising results in sensitivity and specificity.

Statistical analysis

IBM SPSS software (version 20.0; Armonk, NY, US), GraphPad Prism (version 9.0; GraphPad Software), and R (version 4.1.1; R Foundation for Statistical Computing) were used for general statistical analyses. Survival analyses were performed in R (version 4.1.1) using the log‐rank (Mantel-Cox) test to assess differences between groups. Gene‐clinical correlations were evaluated with the Mann-Whitney U and Kruskal-Wallis tests. Unequal and occasionally small group sizes (e.g., 11 metastatic vs. 83 primary samples in cohort 2; 19 vs. 19 in cohort 1) prompted our choice of non-parametric methods, which do not assume normality or equal variances and are more robust for heterogeneous, platform‐derived data than parametric t-tests or ANOVA. A two-sided p-value < 0.05 was considered significant. Fisher’s exact test was applied across all four cohorts to examine gene-specific associations, and Venn diagrams were generated to display commonly significant genes among cohorts.

## Results

Identification of DEGs in BC metastasis (discovery cohort)

**Figure 2:**
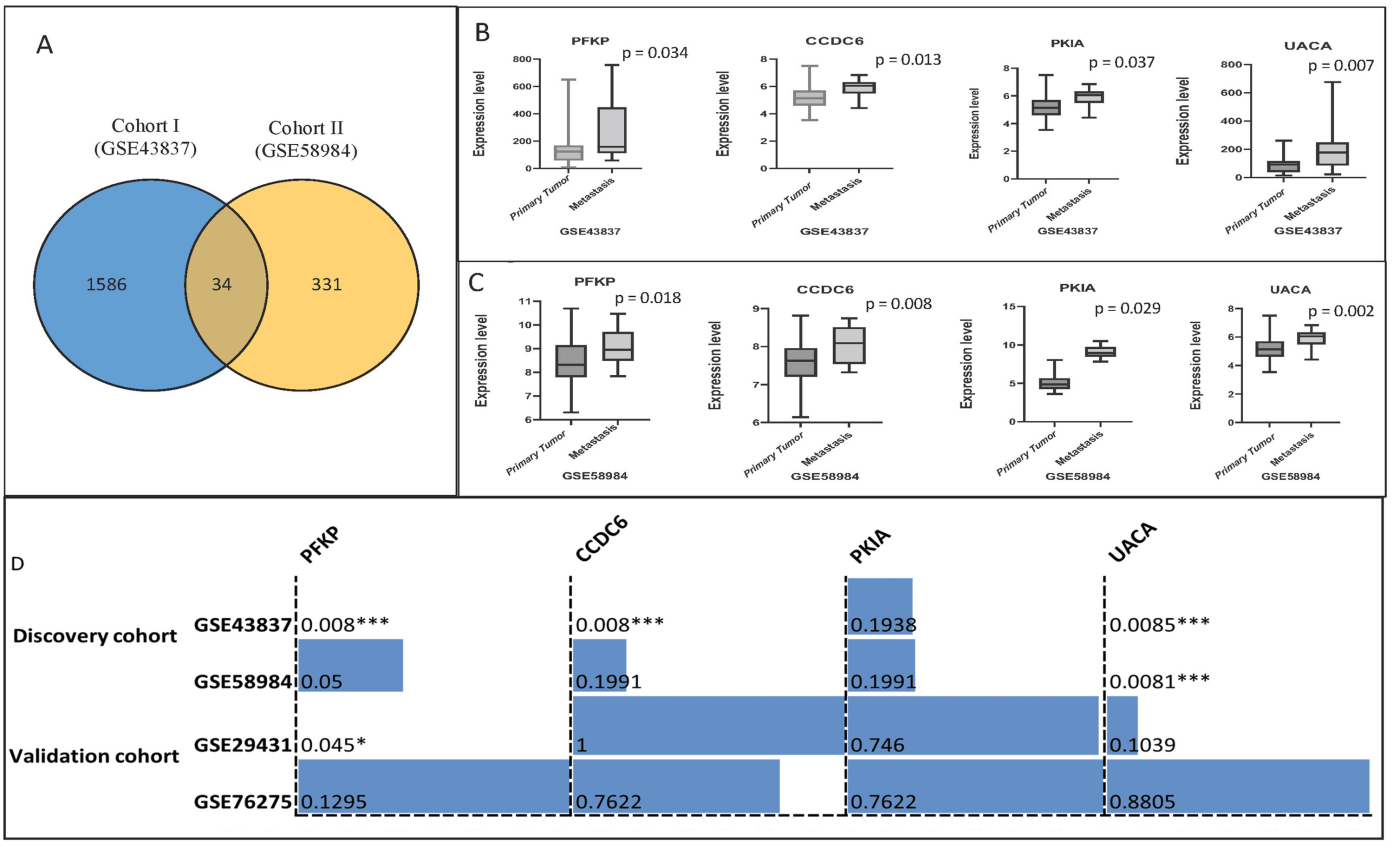
Differentially expressed genes (DEGs) and significant genes and their association with breast cancer. Significant DEGs were defined by more than 1.5 differences: (A) common gene among discovery cohorts 1 & 2. (B, C) Expression profiles of significant common DEGs in primary breast tumor and metastasis among cohorts 1 and 2, respectively. Each graph represents the profile of the relevant gene as indicated. (D) Association of four candidate genes in breast cancer metastasis. The asterisks mark statistically significant associations in each cohort: *p < 0.05; ***p < 0.001. PFKP: phosphofructokinase, platelet isoform; CCDC6: coiled-coil domain containing 6; PKIA: protein kinase inhibitor alpha; UACA: uveal autoantigen with coiled-coil domains and ankyrin repeats; GSE: gene expression series.

Two microarray datasets were selected as discovery cohorts 1 and 2 (GSE43837 and GSE58984), having primary breast tumor expression data and metastasis retrieved from the GEO database to identify metastasis-specific DEGs. We consider the expression profiles with FC (FC > 1.5) of an upregulated gene with p-values < 0.05. We found 365 DEGs in discovery cohort 1 (GSE43837) and 1,620 DEGs in cohort 2 (GSE58984). DEGs are statistically significant between metastatic and localized primary breast tumors (Figure 2A). The details of critical DEGs showing FC expression are shown in Supplemental material 2. We found 34 common genes in both discovery cohorts with significant upregulated and downregulated genes, defined by an FC of 1.5 and a p-value less than 0.05.

Identification of metastasis-related genes and clinicopathological analysis

We found only four common, statistically significant overexpressed genes in metastasis compared to primary breast tumors among the 34 common DEGs in the discovery cohort. These genes include CCDC6, PFKP, PKIA, and UACA. All four genes showed considerable upregulation (p < 0.05) in metastatic tumors compared to primary breast tumors in study cohort 1 (Figure 2B and Supplemental material 3). Consistently, in discovery cohort 2 (GSE58984), these genes showed significant (p < 0.05) upregulation in metastatic tumors (Figure 2C). The Fisher exact test demonstrates that the association for candidate genes also indicates that these genes are positively associated with metastasis except PKIA (Figure 2D). However, we observed the downregulation of PFKP in tumors with positive ER/PR status in cohort 2 (Supplemental material 3). In contrast, the PKIA gene was upregulated in patients with age > 50 years (Supplemental material 3).

PFKP expression profiling in the BC study cohort

**Figure 3:**
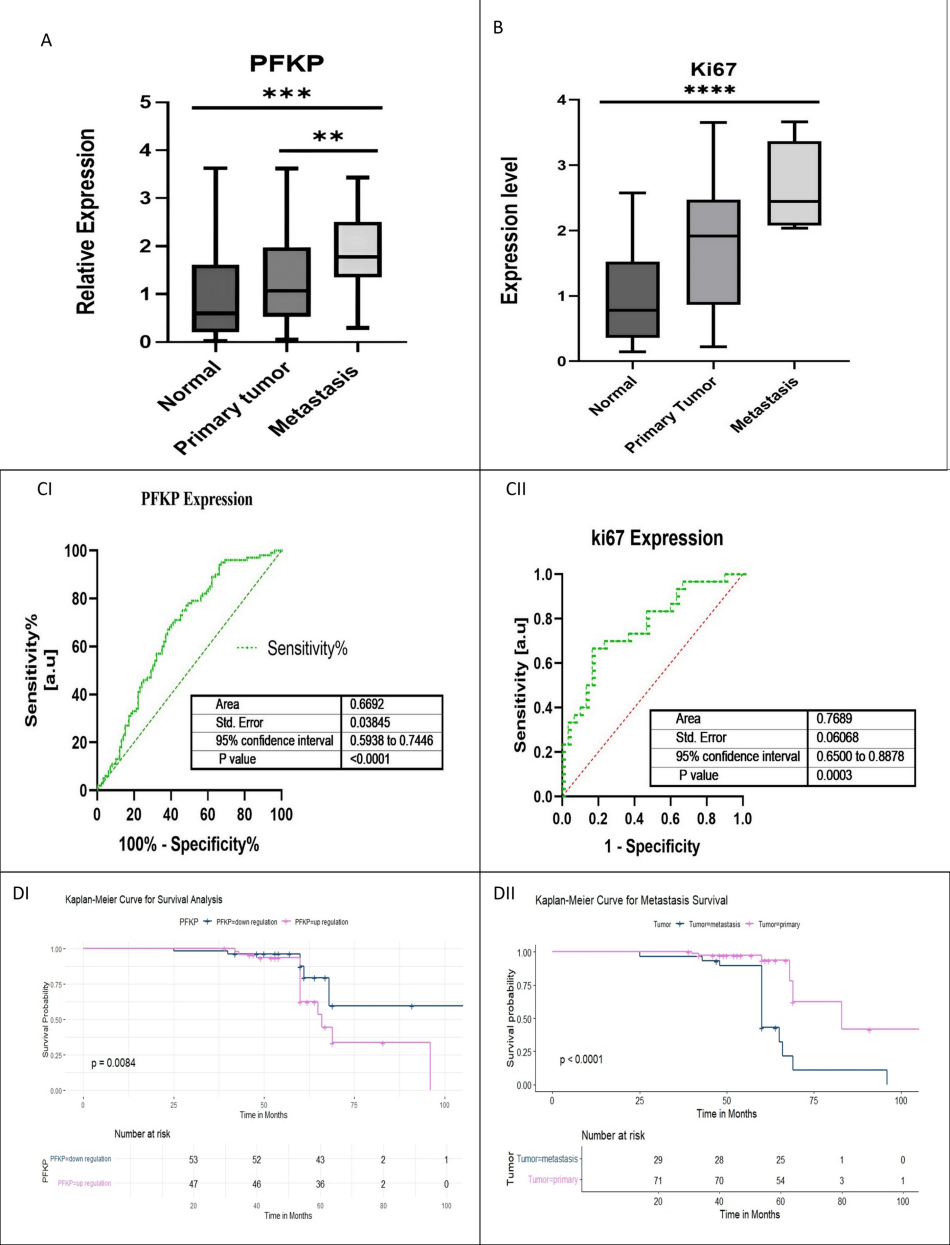
Relative expression profiles of PFKP and Ki67 transcript in breast cancer patients. (A) Fold change expression of the PFKP gene in normal, primary tumor, and metastasis. *** indicates a highly significant increase in PFKP in metastases versus adjacent normal control tissues (ANCTs) (p < 0.001); ** indicates a significant increase in metastases versus primary tumors (p < 0.01). (B) Relative expression of Ki67 was upregulated in metastasis compared to primary tumor and normal as observed in the normal and patient data. **** indicates a very highly significant increase in Ki67 in metastases versus ANCT (p < 0.0001). (CI, CII) ROC curve of PFKP and Ki67 of relative expression demonstrates the significance. (DI, DII) Kaplan-Meier analysis for PFKP expression and primary tumor with metastasis. PFKP: phosphofructokinase, platelet isoform; Ki67: proliferation marker protein; ROC: receiver operating characteristic.

We examined PFKP and Ki67 expression using qRT-PCR in metastasis, primary breast tumors, and ANCTs. We observed PFKP and Ki67 significantly upregulated in metastasis than primary tumor (p < 0.0001) compared to the ANCT cohort (Figure 3A), which highlights its putative involvement in breast carcinogenesis. Therefore, we revealed a significantly higher expression of PFKP and Ki67 (p < 0.0001 and p < 0.0001) genes in metastatic tumors (Figures 3A, 3B). We found a strong association of PFKP expression with metastasis, showing a Pearson Chi-squared value (8.73) with a significant p-value (0.005) (Table 3). Moreover, we observed an association of PFKP expression with Ki67, a biomarker for tumor metastasis, and calculated the Chi-squared value (42.85), showing a highly significant p-value (<0.0001). It signifies that PFKP expression may have potential prognostic roles in cancer pathogenesis. Interestingly, the ROC curve analysis suggests that PFKP may be a good predictor of metastasis, with specificity over 71% (Figures 3CI, 3CII) in the cohort, which is comparable with Ki67 (with specificity over 75%), which means it could be a good predictor of overall prognosis in BC.

**Table 3:** Pearson Chi square for PFKP and Ki67% expression with metastasis. * shows the statistically significant association of Ki67 and metastasis (p < 0.05); ** shows significance at p < 0.01. PFKP: phosphofructokinase, platelet isoform; Ki67: proliferation marker protein.

Fisher’s exact test	PFKP expression	Ki67% expression
Chi square	8.734	6.652
p-value	0.005**	0.025*

Prognostic significance of PFKP expression in BC

Analysis using the Kaplan-Meier method (log-rank test) indicated reduced OS in patients with increased PFKP expression (median OS: 66 months compared to 96 months), showing statistical significance (p = 0.0084) (Figures 3DI, 3DII). In contrast, BC cases featuring primary tumors with metastasis exhibited significantly different expression profiles, with a median OS of 83 versus 60 months (p < 0.00001). The survival statistics and hazard ratios for PFKP expression and primary tumor with metastasis are mentioned in Table 4. Furthermore, metastasis is associated with a significantly poorer survival outcome, highlighting the importance of the early detection and treatment of metastatic disease. These results also support the potential of metastatic status as a prognostic biomarker for patients with BC.

**Table 4:** Survival statistics and hazard ratios for PFKP expression and primary tumor with metastasis. Significant p-value < 0.0001 shows as ****. PFKP: phosphofructokinase, platelet isoform; CI: confidence interval; HR: hazard ratio; coefficient: Cox regression coefficient used to compute hazard ratios; NA: not available; -: value not calculated or not applicable.

Variables	Sample	Events	Median	95% CI	Coefficient	HR	p-value
PFKP downregulation	53	8	NA	68	-	-	0.0084****
PFKP upregulation	47	19	66	60	2.96	2.959	
Primary tumor	71	7	83	69	0.127	-	<0.0001****
Metastasis	29	20	60	60	NA	-	

Validation of metastatic DEGs through external microarray datasets

**Figure 4:**
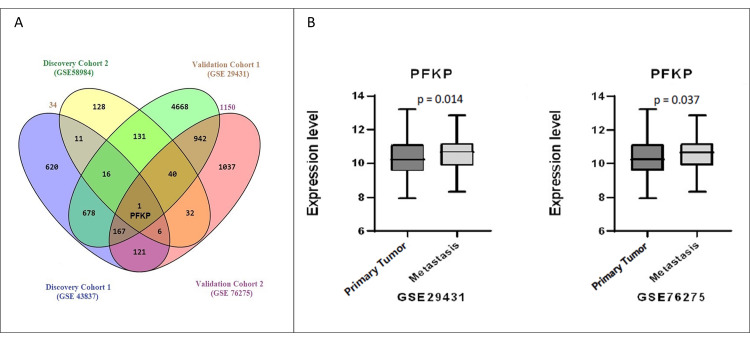
Validation of DEGs in breast cancer metastasis and pathway analysis. (A) Common gene in all four study cohorts. Only PFKP was commonly found upregulated in all cohorts; (B) PFKP was upregulated in metastatic tumors as observed in validation cohorts GSE29431 and GSE76275. PFKP: phosphofructokinase, platelet isoform; GSE: gene expression series; DEGs: differentially expressed genes.

We finalized two microarray GEO datasets to validate the findings mentioned above. We discovered 6,643 DEGs significant among metastatic and primary breast tumors in validation cohort 1 (GSE29431), while 2,346 genes in cohort 2 (GSE76275) (Figure 4A). We observed 1,150 DEGs commonly present in both cohorts. Interestingly, we confirmed only the PFKP gene out of four common genes (CCDC6, PFKP, PKIA, and UACA) of the discovery cohorts among the four significantly upregulated genes. The significant expression of the PFKP gene in metastasis-related tumors compared to primary tumors is shown in Figure 4B. The presence of PFKP in all four datasets highlights its significance in tumor metastasis and may have exciting roles in disease progression.

Additionally, the downregulation of PFKP expression was found significant in ER-positive cells in validation cohort 1 (GSE29431), while we observed PFKP negatively associated with ER/PR/HER2 status of tumors in validation cohort 2 (GSE76275) (Supplemental material 2). PFKP potential highlights as a metastatic marker.

Functional specification of PFKP

To identify the potential function of PFKP in BC metastasis, GSEA was performed in the high- and low-PFKP groups from the GSE29431 and GSE58984 BC cohorts, divided according to the median expression value. We identified positively correlated genes that are significantly enriched in the high PFKP expression group compared to the low PFKP expression group. We detected only four common statistically significant genes, namely, ENO1 (enolase 1), PGM1 (phosphoglucomutase 1), LDHB (lactate dehydrogenase B), and PGK1 (phosphoglycerate kinase 1), significantly overexpressed with PFKP expression in BC patients.

**Figure 5:**
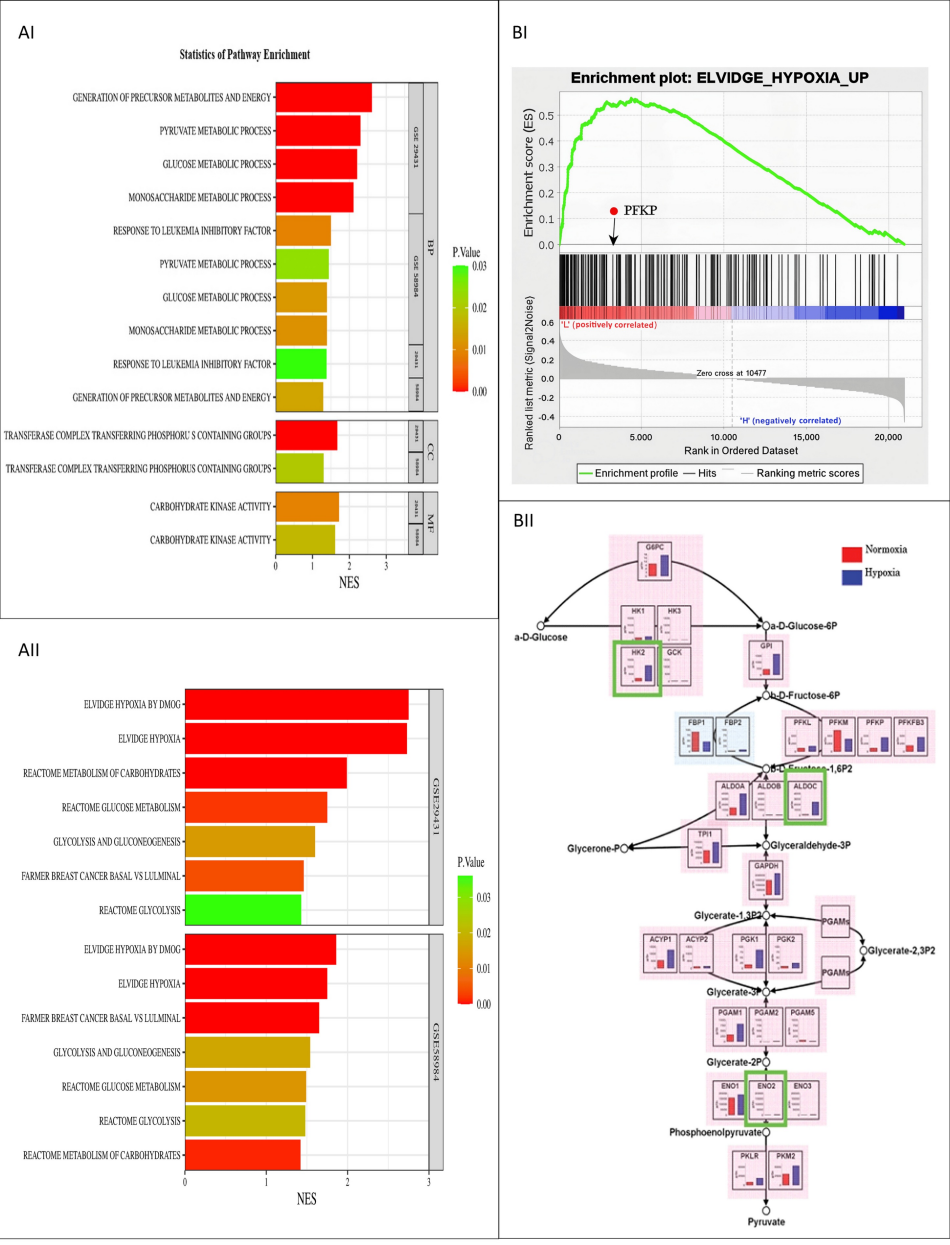
Enrichment analysis. (AI) Bar chart of GO. (AII) Bar chart of significantly enriched PFKP pathways in GSE29431 and GSE58984 obtained from curated genes of Human MSigDB Collections. NES describes the normalized enrichment score. (BI) Gene set enrichment plot for the Elvidge-hypoxia pathway; NES for the Elvidge-hypoxia gene set is 1.54 and p-value 0.009, signifying the involvement of PFKP. (BII) Hypoxia-invoked response of the glycolysis gene network. The expression of glycolysis-enhancing enzymes (masked with pale pink) was upregulated, while that of glycolysis-suppressing enzymes (showed with pale blue) was downregulated. Human genes assigned to the glycolysis pathway map of the KEGG database were selected, and TSS-tag numbers of the corresponding genes were evaluated. Red and blue bars represent TSS-tag ppm in normoxia and hypoxia, respectively (Cytoscape). GO: Gene Ontology; NES: normalized enrichment score; FDR: false discovery rate; PFKP: phosphofructokinase, platelet isoform; MSigDB: Molecular Signatures Database; KEGG: Kyoto Encyclopedia of Genes and Genomes; TSS: transcription start site; GSEA: gene set enrichment analysis.

Further, we identified the significantly enriched pathways with the PFKP expression level. Using GO terms, we performed an over-representation analysis to analyze peculiar pathways associated with these DEGs. Overrepresented pathways in biological processes (BPs), cellular compartments (CCs), and molecular functions (MFs) are shown in Figure 5AI. We found PFKP and co-expressed genes involved in pathways like reactome glycolysis, glucose metabolism, Elvidge hypoxia, and breast carcinogenesis (NES 0 to 1.5, p-value < 0.05) (Figure 5AII).

The gene set enrichment revealed that hypoxia-related genes, including PFKP, were upregulated in these tumors (Figure 5BI). These findings highlight that PFKP is involved in hypoxia-induced changes in glycolysis to meet the altered energy demands of metastatic tumors. Besides, we also performed protein network analysis using Cytoscape software to elucidate underlying putative protein interactions synergistically involved in modulating glycolysis. Figure 5BII highlights the involvement of these genes in mediating single-carbon metabolism under normoxic and hypoxic conditions. Since cancer cells have abnormally high glycolytic flux compared to normal cells, elevated PFKP expression is coherent with the situation to meet energy metabolism demands.

Association of PFKP with glycolysis pathway genes

**Figure 6:**
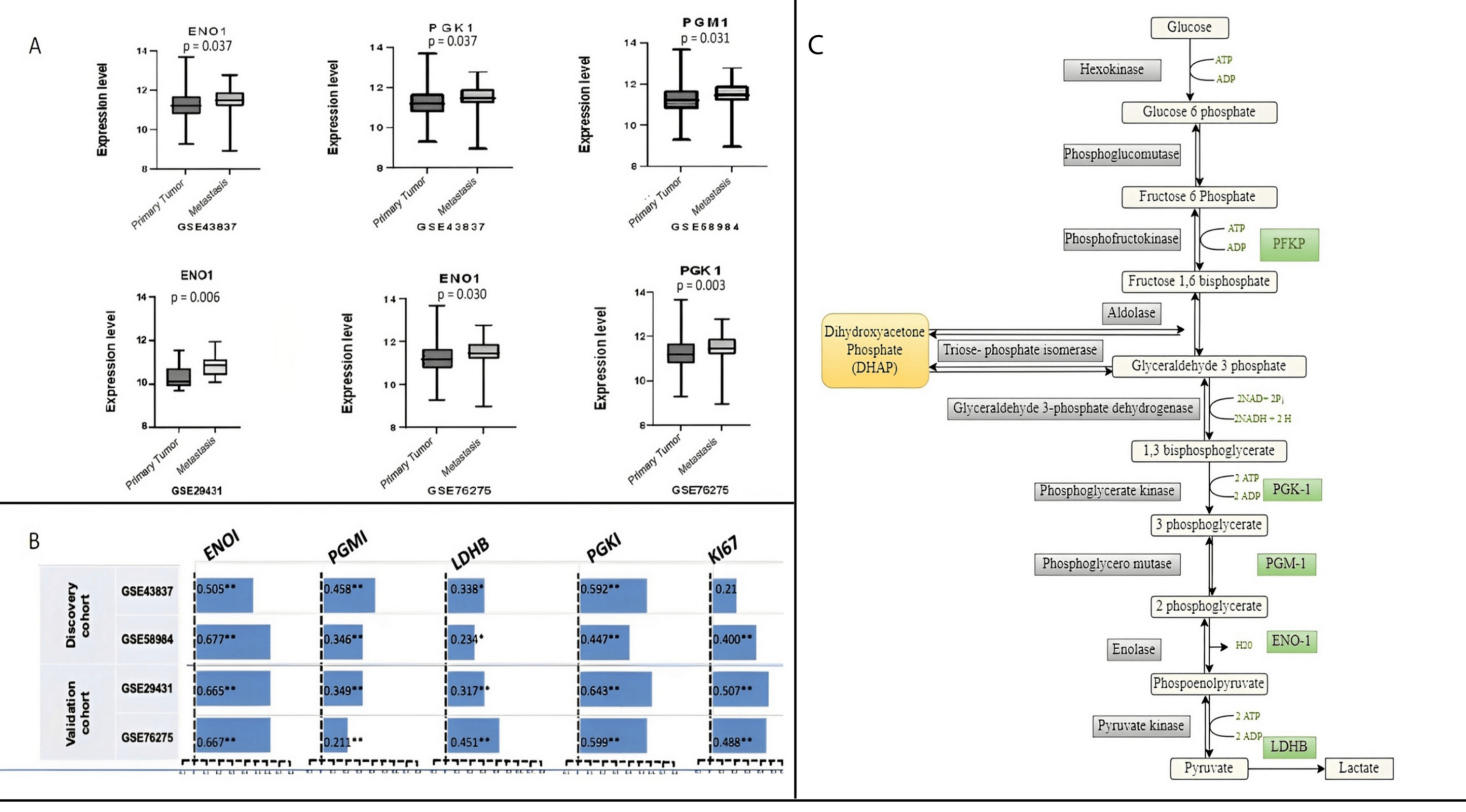
Co-expression and association of various glycolytic genes. (A) PFKP mediated differentially expressed glycolysis genes in breast cancer metastasis. Each graph represents the profile of the relevant gene as indicated; (B) Pearson correlation analysis of PFKP with various glycolysis genes in breast cancer metastasis. Bar chart highlighting the association of given genes with PFKP in tumor metastasis. PFKP was also associated with Ki67. The “blue” bar color represents an association. “*” demonstrates significance at p < 0.05, and “**” indicates significance. (C) Functional specification of PFKP in the glycolysis pathway. This schematic maps the conversion of glucose to lactate through the 10 canonical steps of glycolysis. The rate-limiting enzyme phosphofructokinase-1 (PFK1) is highlighted, converting fructose-6-phosphate to fructose-1,6-bisphosphate. Downstream enzymes PGK1, PGM1, ENO1, and LDHB are boxed in green, denoting each as a significant differentially expressed gene (DEG) correlated with PFKP in our cohorts. Figure (C) was created in-house using Draw.io, adapted from the Kyoto Encyclopedia of Genes and Genomes (KEGG) Pathway Database, Glycolysis/Gluconeogenesis–Homo sapiens (hsa00010) [15]. PFKP: phosphofructokinase, platelet isoform; ENO1: enolase 1; PGM1: phosphoglucomutase 1; LDHB: lactate dehydrogenase B; PGK1: phosphoglycerate kinase 1; KEGG: Kyoto Encyclopedia of Genes and Genomes; Ki67: proliferation marker protein.

We also identified the co-expression patterns of glycolysis pathway genes associated with PFKP. We detected four glycolysis pathway genes, namely, ENO1 (enolase 1), PGM1 (phosphoglucomutase 1), LDHB (lactate dehydrogenase B), and PGK1 (phosphoglycerate kinase 1), significantly co-expressed with PFKP in BC patients. Except for LDHB, the rest of the three genes were differentially expressed in the discovery and validation cohorts. The expression profiles of these metastases associated with PFKP co-expressed genes are shown in Figure 6A.

Further, we evaluate the association of glycolysis pathway genes with PFKP expression. These genes are associated positively with PFKP overexpression in all study cohorts (Figure 6B). Pathway analysis revealed that the deregulated expression of PFKP may perturb the expression of vital glycolytic genes (PGK1, PGM1, ENO1, and LDHB) represented in Figure 6C. It suggests that PFKP is involved in BC metastasis by mediating the gene expression of the genes involved in the glycolysis pathway.

## Discussion

The key finding of the current study is that PFKP is upregulated in all cohorts (discovery and validation) regarding tumor, control, and metastasis. The relative expression of PFKP and Ki67 was upregulated in primary tumors and metastasis, according to the patient data. ROC analysis offers predictive values for PFKP and Ki67. GSEA revealed a glycolysis pathway, common in the cohorts. Metastasis is associated with a significantly poorer survival outcome, highlighting the importance of early detection. It proves that the therapeutic intervention of the PFKP may be used as a predictive biomarker.

Alterations in the tumor microenvironment are the key to triggering a neoplastic transformation in healthy tissues and organs. Profound metabolic differences occurred in tumor cells to meet their metabolic requirements, i.e., increased glucose uptake and high energy demands. Tumor tissues lyse glucose through aerobic glycolysis (Warburg effect). The Warburg effect is the tendency of tumor cells to take up glucose and secrete lactate even in the presence of oxygen and fully functional mitochondria. Due to this effect, increased cytoplasmic glucose absorption is a hallmark of malignant tissues, while the mitochondrial respiration rate remains unchanged [16]. Thus, altering the expression of the glycolysis pathway and genes in the hypoxia condition may offer tumor cells an advantage in growing in a multicellular environment.

Phosphofructokinase-1 (PFK1) is a vital rate-limiting enzyme in cellular respiration and oxidative metabolism. It catalyzes the phosphorylation of fructose-6-phosphate (F6P) to fructose-1,6-bisphosphate (F1,6BP) [17]. Any dysregulation in its expression may perturb cellular energy demands. There are three isoforms of PFK1, i.e., PFKP in platelets, PFKM specific to muscle, and PFKL in the liver. PFKP describes its high prevalence in various cancers [18], including glioblastoma and BC [19]. The current study also presents PFKP as a vital target gene promoting metastasis in BC. Since cancer cells have abnormally high glycolytic flux compared to normal cells, elevated PFKP expression is coherent with the situation to meet energy metabolism demands. Such elevated levels of PFKP were explained previously as a prevalent feature of malignant tissues [20,21]. Studies show that PFKP expression increases in AML patients more than in healthy controls, especially in the subtypes with poor cytogenetic risk [22]. A recent study demonstrated that in intrahepatic cholangiocarcinoma, the overexpression of PFKP and the activation of AMPK resulted in IDH1 mutation in normal biliary cells. High PFKP expression is observed in patients with an IDH1 mutation [23].

Moreover, PFKP participates in cancer metastasis by regulating lactate production [24]. However, its involvement in tumor metastasis remained elusive. Consistent with these reports, our findings corroborate a putative role of PFKP in tumor metastasis. Moreover, high PFKP expression is associated with poor prognosis and patient survival outcomes [25]. In the current study, we further investigated the network of genes related to PFKP in promoting tumor metastasis. Four other glycolytic genes, ENO1, PGM1, LDHB, and PGK19, were upregulated in metastatic tumors compared to non-metastatic tumors. PFKP-mediated expression of other glycolytic gene conditions is imperative to support the tumor microenvironment to promote metastasis. It is worth mentioning that several reports have shown elevated levels of these gluconeogenesis-related genes, especially ENO1, PGK1, and PKM, in tumor tissues [26]. Similarly, the combined effects of mutations in PFKP and ENO1 are associated with worse patient survival outcomes [27].

The evidence proves that cancer is a metabolic disorder; deregulated glycolysis is shown to hamper anticancer drug activities in cancer patients. Aberrant glycolysis (hypoxic conditions) plays a significant role in drug-resistant cancer cells [28,29]. Several glycolytic enzymes play a role in drug resistance induction, including hexokinase (HK2), pyruvate kinase (PKM2), pyruvate dehydrogenase (PDH) complex, and lactate dehydrogenase (LDH). However, the problem of chemoresistance in cancer prognosis remained an unsolved riddle. As a key rate-limiting enzyme in cancer glycolysis, PFKP is appearing as a potential anticancer drug target. Its ability to arrange other glycolytic enzymes can be crucial in patients' overall prognosis and survival [30]. However, the efficacy and clinical applicability of anticancer agents targeting PFKP remain under investigation. A limitation of our study is the absence of functional laboratory experiments such as PFKP knockdown or overexpression in cell lines or animal models with subsequent assessment of migration, invasion, proliferation, and other cancer-related behaviors to establish a causal role for PFKP in BC metastasis.

## Conclusions

Current data suggest that PFKP, a rate-limiting enzyme in glycolysis, is involved in BC metastasis. Its elevated expression also influences other glycolytic genes that initiate a metastatic cascade, including ENO1, PGM1, LDHB, and PGK1. ROC curve analysis also suggests that PFKP could be a good predictor of overall prognosis in BC patients and is applicable to predict metastatic potential at initial stages. Thus, regulating PFKP expression could prevent tumors' metastatic fate. Despite advancements in early detection, improvement in the OS of patients remained grim due to the unavailability of an effective strategy to predict the metastatic and relapse potential of primary tumors.

## Appendices

Supplemental material 1 

**Table 5:** List of primers. The forward and reverse primer sequences used for quantitative real-time polymerase chain reaction (qRT-PCR) validation of selected genes in breast cancer samples. Primers were designed to target specific gene transcripts, including PFKP, Ki67 (a proliferation marker), and β-actin (used as a housekeeping control).

Gene	Forward primer sequence	Reverse primer sequence
PFKP	CTGGGTGTTCCTTCCAGAAT	GTGACGACAAGCTCTTTGATTT
Ki67	TCCTTTGGTGGGCACCTAAGACCTG	TGATGGTTGAGGCTGTTCCTTGATG
β-Actin	CACTCTTCCAGCCTTCCTTC	TGATCTCCTTCTGCATCCTG

Supplemental material 2 

**Table 6:** Identification of differentially expressed genes (DEGs) and their expression. The expression profiles of significantly upregulated genes in metastatic breast cancer samples compared to primary tumors across two datasets (GSE43837 and GSE58984). The geometric mean intensities of each gene are reported for both classes. Fold change values were calculated, and all listed genes (PFKP, PKIA, UACA, and CCDC6) showed consistent upregulation in metastasis.

Dataset	Geom mean of intensities in class 1 primary breast tumor	Geom mean of intensities in class 2 metastasis	Fold change	Gene	Expression of the gene in class 2 metastasis
GSE43837	18.98	43.54	2.293	PKIA-75209	Upregulation
	51.45	124.24	2.414	CCDC6-288862	Upregulation
	125.75	197.02	1.566	PFKP-11321600	Upregulation
	76.61	114.74	1.5	UACA-53810	Upregulation
GSE58984	18.6	34.67	1.863	PKIA-226864	Upregulation
	177.55	250.52	1.410	CCDC6-204716	Upregulation
	267.38	426.13	1.593	PFKP-201037	Upregulation
	166.15	221.2	1.274	UACA-223279	Upregulation
	28.08	45.85	1.632	UACA-238868	Upregulation

Supplemental material 3 

**Table 7:** Clinical analysis of significant common genes in the discovery and validation cohorts. Gene expression associations between four common genes (PFKP, UACA, PKIA, and CCDC6) and selected clinical parameters across four independent breast cancer cohorts (GSE43837, GSE58984, GSE29431, and GSE76275). Each row lists a single gene expression event related to a clinical characteristic within a dataset. Upregulation indicates increased expression of the gene in the specified subgroup. Downregulation indicates decreased expression in that subgroup. Clinical parameters include the metastasis status (M0 vs. M1), age category (>50 years), hormone receptor status (ER-positive, PR-positive), tumor grade (T3 & T4), and Ki67% proliferation index (>20%). PFKP: phosphofructokinase, platelet isoform; UACA: uveal autoantigen with coiled-coil domains and ankyrin repeats; PKIA: protein kinase inhibitor alpha; CCDC6: coiled-coil domain containing 6; ER: estrogen receptor; PR: progesterone receptor; Ki67: cellular proliferation marker.

Dataset	Clinical parameters	Gene	Expression
GSE43837	Metastasis	PFKP	Upregulation
		UACA	
		CCDC6	
		PKIA	
GSE58984	Metastasis	PFKP	Upregulation
		UACA	
		CCDC6	
		PKIA	
GSE29431	Metastasis	PFKP	Upregulation
GSE76275	Metastasis	PFKP	Upregulation
GSE58984	Age > 50	PKIA	Upregulation
		UACA	Downregulation
	ER-positive	PFKP	Downregulation
GSE29431	ER-positive	PFKP	Downregulation
GSE76275	ER-positive	PFKP	Downregulation
		PKIA	Upregulation
		UACA	Upregulation
GSE58984	PR-positive	PFKP	Downregulation
GSE29431	PR-positive	PFKP	Downregulation
GSE76275	PR-positive	PKIA	Upregulation
		UACA	Upregulation
	Tumor grades T3 & T4	PFKP	Upregulation
		UACA	Downregulation
GSE29431	Ki67% > 20	UACA	Downregulation
